# A Loop-Mediated Isothermal Amplification (LAMP) Assay Specific to *Trichomonas tenax* Is Suitable for Use at Point-of-Care

**DOI:** 10.3390/microorganisms10030594

**Published:** 2022-03-10

**Authors:** Maurice A. Matthew, Jevan Christie, Nawu Yang, Chaoqun Yao

**Affiliations:** 1 Department of Biomedical Sciences, Ross University School of Veterinary Medicine, P.O. Box 334, Basseterre 00334, Saint Kitts and Nevis; MMatthew@rossvet.edu.kn (M.A.M.); NawuYang@students.rossu.edu (N.Y.); 2 One Health Centre for Zoonosis and Tropical Veterinary Diseases, Ross University School of Veterinary Medicine, P.O. Box 334, Basseterre 00334, Saint Kitts and Nevis; jevanchristie@gmail.com; 3 Department of Clinical Sciences, Ross University School of Veterinary Medicine, P.O. Box 334, Basseterre 00334, Saint Kitts and Nevis

**Keywords:** loop-mediated isothermal amplification (LAMP), *Trichomonas tenax*, sensitivity, specificity, periodontal disease

## Abstract

*Trichomonas tenax* is a flagellated protozoan that inhabits the human and canine oral cavity in patients with poor oral hygiene and periodontal disease. The loop-mediated isothermal amplification (LAMP) assay could provide clinicians with a quick, cheap and reliable diagnostic test used for the detection of *T. tenax* in various settings. In this study, we aimed to develop a LAMP assay that can detect *T. tenax* with high sensitivity and specificity. A set of LAMP primers were specifically designed to detect the ITS and 5.8S rRNA gene of *T. tenax*. The newly developed LAMP assay was 1000 times more sensitive than conventional PCR. The limit of detection of the LAMP assay was 10 fg of genomic DNA, or 0.2–1 cell. Moreover, the LAMP assay was specific, resulting in no cross-reaction even with a closely related protozoan *T. vaginalis* or other microorganisms (*Streptococcus pyogenes*, *Staphylococcus aureus*, *Escherichia coli*, *Enterococcus faecalis*, and *Candida albicans*) used. The present LAMP assay can be performed directly without prior DNA extraction, making the assay an easy, fast, cheap, specific and sensitive diagnostic tool for the detection of *T. tenax* at the point-of-care of both medical and veterinary clinics in developed and developing countries.

## 1. Introduction

*Trichomonas* *tenax* is a flagellated protozoan that inhabits the human and canine oral cavity in patients with poor oral hygiene and periodontal disease [1,2,3,4]. The protozoan belongs to the same genus as *T. vaginalis*, a human pathogen of great medical importance worldwide [5]. In addition, *T. tenax* has also been detected in other tissues and organs of the body such as the respiratory tract and lymph nodes [6,7,8,9]. *T. tenax* has been reported in humans since the 1960s [4] and has been specifically connected to human periodontal disease by a systematic review and meta-analysis of 65 qualified publications [10]. In a prospective study carried out in Jordan recently, it has been found that the prevalence of *T. tenax* among healthy, gingivitis and periodontitis groups is 3.2%, 5.7% and 25.6%, respectively. In the periodontitis group, the prevalence of *T. tenax* among individuals with generalized or localized conditions is 19.6% and 0.0%, respectively (*p* = 0.039). Furthermore, the odds ratios with *T. tenax* infection between periodontitis and healthy groups, and between periodontitis and gingivitis groups are 4.7 (95% CI: 1.0–21.8, *p* = 0.045) and 7.2 (95% CI: 1.5–33.8, *p* = 0.013), respectively [11]. In a study of 92 samples of canine plaque using touchdown PCR and next generation sequencing, the proportion of *Trichomonas* sp. among health, gingivitis, early and severe periodontitis is 3.5%, 2.8%, 6.1% and 35.0%, respectively [12]. “Despite its relatively frequent association with periodontal diseases, *T. tenax* has not received enough attention as a putative pathogen, mainly due to the lack of simple detection methods” [1]. Over the past years researchers have been using PCR to detect and identify *T. tenax*, and update the prevalence of this parasite from traditional diagnostic methods previously used which included culture and microscopy [1,2,3,4,12,13]. PCR has a higher sensitivity compared to culture and microscopy [1,2,3,4,13]. Despite these advantages, PCR assays still require expensive laboratory equipment, reagents, highly trained professionals and it can be time-consuming to achieve accurate results [14].

Over the last two decades, a method termed loop-mediated isothermal amplification (LAMP) has established itself as a method that amplifies DNA with high specificity, efficiency and rapidity under isothermal conditions [15]. LAMP uses a DNA polymerase with strand displacement activity and a set of 4–6 primers that amplifies DNA under isothermal conditions, thus incubation can be done in a heat block or water bath [16]. Numerous studies have concluded that LAMP is a rapid, cost-effective, sensitive and specific nucleic acid amplification test that can be used for the diagnosis of various infectious diseases especially in developing countries [15,16,17,18]. For example, it has been used to detect protozoa such as *Toxoplasma* sp., *Trypanosoma* sp., *Cryptosporidium* sp., *Entamoeba histolytica*, *Tritrichomonas foetus* and *T. vaginalis* [18]; bacteria such as *Mycobacterium tuberculosis* [19], *Yersinia enterocolitica* [17] and *Enterococcus faecalis* [20]. In this study, we aimed to develop a LAMP assay that can detect *T. tenax* with high sensitivity and specificity. This newly developed LAMP assay should provide a quick, cheap, easy and reliable method used for the detection of *T. tenax* in both developed and developing countries that can be used as a point-of-care test in human and veterinary medicine.

## 2. Materials and Methods

### 2.1. Cells and Culture

*T. tenax* strain Hs-4:NIH obtained from the American Type Culture Collection (ATCC, Manassas, VA, USA) was axenically grown at 35 °C for 5 days in a modified Diamonds’ medium as previously described [21,22]. The medium was supplemented with 10% heat-inactivated horse serum (Sigma-Aldrich, St. Louis, MO, USA) and penicillin and streptomycin (ThermoFisher, Waltham, MA, USA). Cell density was monitored daily using a hemocytometer (Hausser Scientific, Horsham, PA, USA) [23]. Genomic DNA was extracted from cultured cells using QIAGEN DNeasy Blood and Tissue Kit (QIAGEN, Hilden, Germany) according to the manufacturer’s instructions and was used to optimize and assess analytical sensitivity during the development of the LAMP assay. Bacteria including *Streptococcus pyogenes*, *S. aureus*, *Escherichia coli*, *Enterococcus faecalis* and fungi such as *Candida albicans* were also cultured using sheep-blood agar (BD BBL™, Franklin Lakes, NJ, USA) under aerobic conditions at 37 °C for 24 h. *Trichomonas vaginalis* cells that originated from *T. vaginalis* positive urine samples diagnosed by microscopy were a gift from the Diagnostic Lab of the Joseph N. France General Hospital in St. Kitts and Nevis. These samples that were normally destroyed right after diagnosis were given to us without any patient information. They were only used to assess the analytical specificity of the LAMP assay since *T. vaginalis* is closely related to *T. tenax*.

### 2.2. LAMP Reaction

The LAMP primers targeting the ITS and 5.8S rRNA gene of *T. tenax* (GenBank Accession No. U86615) were designed using the software Primer explorer V.5 (http://primerexplorer.jp (accessed on 28 February 2020). These include FIP and BIP, F3 and B3, LF, and LB (Table 1). Multiple sequence alignment using software CLUSTAL 1.2.4 (Clustal Omega; https://www.ebi.ac.uk/Tools/msa/clustalo/ (accessed on 28 February 2020) was carried out on the closely related protozoan *T. vaginalis*, to check for specificity.

For optimization of the LAMP assay, MgSO_4_ concentration, temperature and reaction time were assessed. To optimize the MgSO_4_ concentration, 2, 4, 6, 8 and 10 mm MgSO_4_ were used. To optimize the temperature, reactions were carried out at 50, 53, 55, 58, 60, 63 or 65 °C. For time optimization, 15, 30, 45, 60 and 75 min were used. These were all performed in Mastercycler^®^ Nexus Gradient Cyclers (Eppendorf, Enfield, CT, USA).

The optimized LAMP reactions for the assay were performed in a 25 µL reaction mixture containing the following reagents: 0.8 µm FIP, 0.8 µm BIP, 0.4 µm LF, 0.4 µm LB, 0.2 µm F3, 0.2 µm B3, 4 mm MgSO_4,_ 1 m betaine, 0.4 mm dNTPs, 1X thermopol buffer [20 mm Tris-HCl, 10 mm (NH4)_2_SO_4_, 10 mm KCl, 2 mm MgSO_4_, 0.1% Triton X-100 pH 8.8], 6 U of Bst polymerase large fragment (New England Biotechnologies, New Haven, CT, USA), 2 µL of template DNA and nuclease free water. Reactions were performed at 60 °C for 60 min in the Mastercycler^®^ Nexus Thermal Cyclers (Eppendorf, Enfield, CT, USA).

The amplified products were visualized using gel electrophoresis and SYBR Green I (Thermo Fisher Scientific, Waltham, MA, USA). For gel electrophoresis, 10 µL of the product was separated on 1% agarose gel containing 0.5 mg/mL ethidium bromide with a 100 bp ladder of DNA marker (Invitrogen, Carlsbad, CA, USA). For detection using SYBR Green I, 1 µL of SYBR Green I was added to 10 µL of the product and examined under UV light. Fluorescent green color is an indication of a positive reaction and orange color is an indication of a negative reaction.

### 2.3. PCR Reaction

Primers F3 and B3 shown in Table 1 were used in the PCR. The PCR was carried out in a 25 µL reaction mixture containing the following reagents: 2 µL template DNA, 1X *Taq* buffer with KCl, 0.2 mm dNTPs, 3 mm MgCl_2_, 1 µm F3, 1 µm B3, 2.5 U of *Taq* DNA polymerase (Qiagen, Germantown, MD, USA), and nuclease-free water. PCR was performed in Mastercycler^®^ Nexus Gradient Cyclers. The thermal cycle was optimized for annealing temperature as follows: initial denaturation for 5 min at 95 °C, 35 cycles of denaturation for 1 min at 95 °C, annealing at 53 °C for 30 s, elongation at 72 °C for 1 min and a final step at 72 °C for 5 min.

### 2.4. Limit of Detection and Specificity

To determine the limit of detection (LD) of the LAMP assay, a 10-fold serial dilution of *T. tenax* genomic DNA in Tris-EDTA (TE) buffer without a carrier, ranging from 50 ng/μL to 5 pg/μL was used. The results were compared to conventional PCR using the LAMP F3 and B3 primers. Analytical specificity of the newly developed LAMP assay was evaluated using a close relative (*T. vaginalis*), along with other microorganisms (*S. pyogenes*, *S. aureus*, *E. coli*, *E. faecalis* and *C. albicans*). Negative and positive controls using water and *T. tenax* genomic DNA, respectively, were included. Each experiment was run three times for repeatability and reproducibility.

### 2.5. Analytical Sensitivity of Spiked Samples

Cultured *T. tenax* cells were used to investigate the limit of detection of the spiked samples. They were diluted in canine saliva, TE buffer or phosphate buffered saline (PBS) in serial dilutions from 2 × 10^5^ to 2 × 10^−1^ cells. These cells were subjected to boiling at 100 °C for 30 min followed by cooling down to 4 °C on ice. Alternatively, similar prepared spiked samples were subjected to no pre-treatment at all and were directly used to perform LAMP. Positive and negative controls using *T. tenax* genomic DNA and water, respectively, were included. The experiment was run three times for repeatability and reproducibility.

### 2.6. Analysis of Canine Samples

LAMP was performed on eight oral swabs that were collected from client owned pet dogs that presented to the RUSVM Veterinary Clinic. These swabs were subjected to culture up to 5 days as mentioned above for the presence of *T. tenax*. The cells were centrifuged, collected and stored at −80 °C until use. To perform LAMP on these samples, the frozen cells were taken by a sterile pipette-tip without thawing and immediately suspended in PBS for quantification. The sample was applied directly to perform LAMP without DNA extraction or boiling after resuspension in TE buffer followed centrifugation. In total, 2 cells per reaction were used to perform LAMP. Positive and negative controls using *T. tenax* genomic DNA and water, respectively, were included. The experiment was run three times for repeatability and reproducibility.

## 3. Results

### 3.1. Development of LAMP Assay

**Figure 1:**
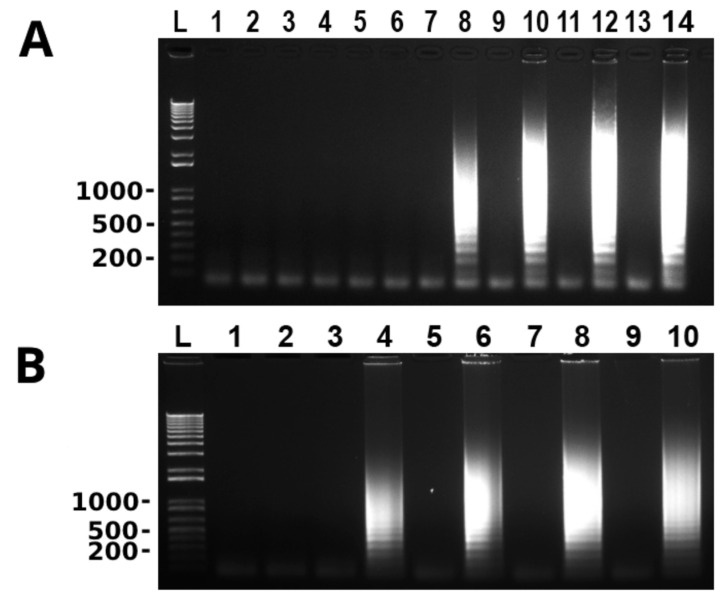
Optimization of LAMP assay for the detection of *T. tenax*. Odd and even lanes are without and with *T. tenax* genomic DNA (100 ng), respectively. (**A**) Temperature optimization of LAMP reaction: lanes 1 and 2, lanes 3 and 4, lanes 5 and 6, lanes 7 and 8, lanes 9 and 10, lanes 11 and 12 and lanes 13 and 14 are at 50, 53, 55, 58, 60, 63 and 65 °C, respectively. (**B**) Time optimization of LAMP reaction: lanes 1 and 2, lanes 3 and 4, lanes 5 and 6, lanes 7 and 8 and lanes 9 and 10 are at 15, 30, 45, 60 and 75 min, respectively. L: 100 bp DNA ladder marker. One of three repeats is shown.

Reaction temperature, MgSO_4_ concentration and time were optimized during the development of the LAMP assay. To optimize temperature, the reaction was incubated at 50, 53, 55, 58, 60, 63, and 65 °C, respectively, for 60 min. Amplification took place at 58, 60, 63 and 65 °C, and 60 °C was chosen as the optimal temperature based on the criteria of maximum LAMP amplification at the lowest temperature in order to extend the enzyme half-life as long as possible (Figure 1A). The MgSO_4_ concentration was optimized by incubation reaction using 2, 4, 6, 8 and 10 mm MgSO_4_, respectively. The optimal MgSO_4_ concentration selected for the assay was 6 mm (data not shown). To optimize the reaction time the reaction was incubated at 15, 30, 45, 60 and 75 min, respectively. Amplification was seen at 30 min and beyond, and the optimal time chosen for the assay was 60 min (Figure 1B). Therefore, all LAMP reactions from this point forward were performed at 60 °C using 6 mm MgSO_4_ for 60 min.

### 3.2. Newly Developed LAMP Assay Is More Sensitive Than PCR

**Figure 2:**
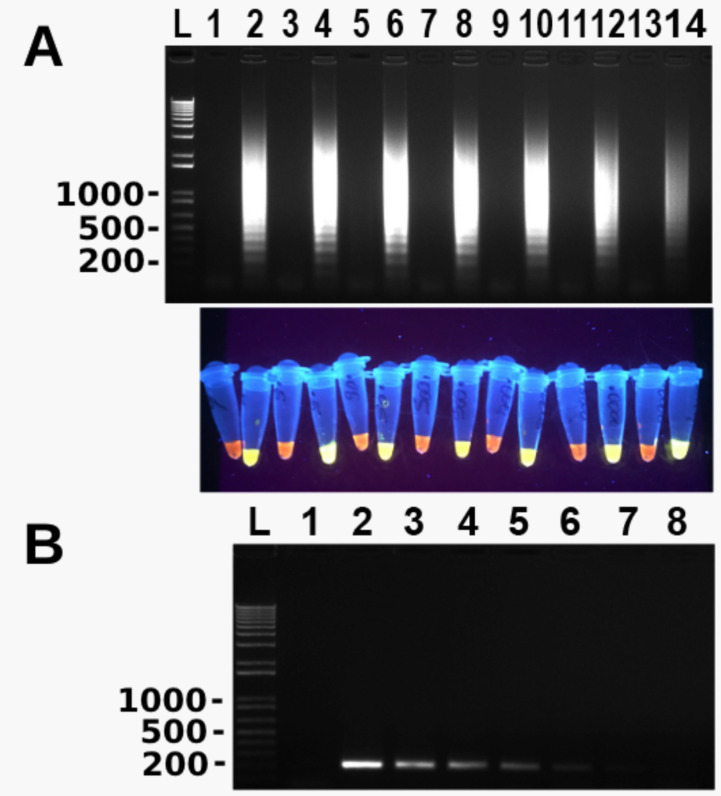
Limit of detection of *T. tenax* by LAMP. Odd and even lanes are without and with serially diluted *T. tenax* genomic DNA, respectively. (**A**) Lanes 1 and 2, lanes 3 and 4, lanes 5 and 6, lanes 7 and 8, lanes 9 and 10, lanes 11 and 12 and lanes 13 and 14 are at 10, 1, 0.1, 0.01, 0.001, 0.0001, 0.00001 and 0.000001 ng, respectively. LAMP results are detected by gel electrophoresis (top panel) and SYBR Green I with UV illumination (bottom panel). (**B**) Limit of detection of conventional PCR: lane 1: negative control with water and lanes 2–8: 100, 10, 1, 0.1, 0.01, 0.001 and 0.0001 ng of DNA, respectively. PCR results are detected by gel electrophoresis. L: 100 bp DNA ladder marker. One of three repeats is shown.

To determine the LD of the newly developed LAMP assay, a 10-fold serial dilution of *T. tenax* genomic DNA with the original concentration of 50 ng/µL was used in LAMP and conventional PCR. The results of both assays were compared. As shown in Figure 2, LD of the LAMP assay was 10 fg *T. tenax* genomic DNA whereas LD of conventional PCR was 10 pg *T. tenax* genomic DNA. The latter was 1000 times higher than the former.

### 3.3. Newly Developed LAMP Assay Is Specific

**Figure 3:**
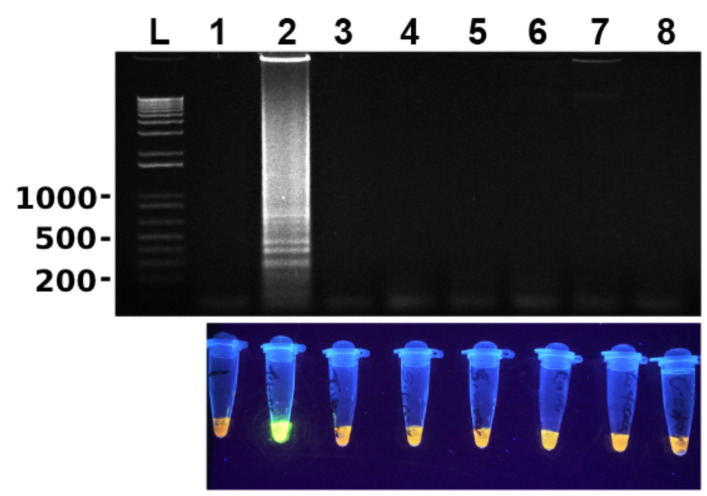
Newly developed LAMP assay is highly specific. Lane 1: no DNA (negative control with water); lanes 2–8: containing 2 µL genomic DNA of various microbes. Lane 2: *T. tenax* (5 ng/µL); lane 3: *T. vaginalis* (137 ng/µL); lane 4: *S. pyogenes* (74ng/µL); lane 5: *S. aureus* (160 ng/µL); lane 6: *E. coli* (52 ng/µL); lane 7: *E. faecalis* (70 ng/µL); and lane 8: *C. albicans* (60 ng/µL). LAMP results are detected by gel electrophoresis (**top panel**) and SYBR Green I with UV illumination (**bottom panel**). L: 100 bp DNA ladder marker. One of three repeats is presented.

To determine the specificity of the newly developed LAMP assay, genomic DNA of several microbes including *T. vaginalis*, *S. pyogenes*, *S. aureus*, *E. coli*, *E. faecalis* and *C. albicans* were used along with that of *T. tenax*. As shown in Figure 3, LAMP did not amplify any DNA of these microbes including closely related *T. vaginalis*. These results clearly show that this LAMP assay is highly specific to *T. tenax*.

### 3.4. Newly Developed LAMP Assay Can Detect T. tenax without Prior DNA Extraction

**Figure 4:**
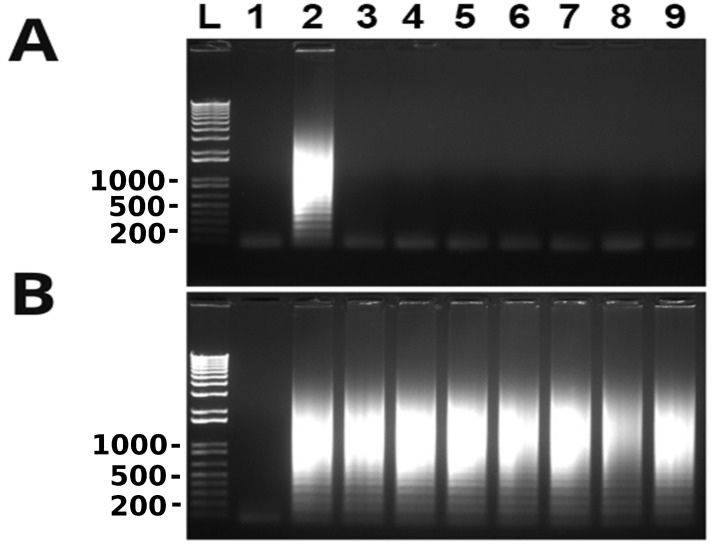
Detection of *T. tenax* without DNA extraction by newly developed LAMP assay. Serially diluted samples of *T. tenax* cells in PBS (**A**) or TE buffer (**B**) boiled at 100 °C for 30 min: lanes 1 and 2: negative and positive controls without and with *T*. *tenax* genomic DNA (100 ng/µL); lanes 3–9: containing 2 × 10^5^, 2 × 10^4^, 2 × 10^3^, 2 × 10^2^, 2 × 10^1^, 2 × 10^0^ and 2 × 10^−1^ cells, respectively. L: 100 bp DNA ladder maker. One of three repeats is presented.

It has been reported that LAMP works in some microbes even without prior DNA isolation such as *T*. *vaginalis* [16]. We probed this possibility with *T. tenax*. Cultured *T. tenax* cells were diluted in PBS or TE buffer in a 10-fold serial dilution. The diluted cells were boiled at 100 °C for 30 min and then used directly in LAMP after being cooling down to 4 °C. As shown in Figure 4, the newly developed LAMP assay detected *T. tenax* in TE buffer to as few cells as 0.2–1 cell per reaction (Figure 4B). In contrast, LAMP reaction was negative even with as many as 2 × 10^5^ *T. tenax* cells in PBS (Figure 4A), indicating that either PBS directly inhibits LAMP reaction or PBS does not release genomic DNA after boiling of cells.

### 3.5. Direct Detection of T. tenax Spiked Saliva and Clinically Collected Canine Samples

**Figure 5:**
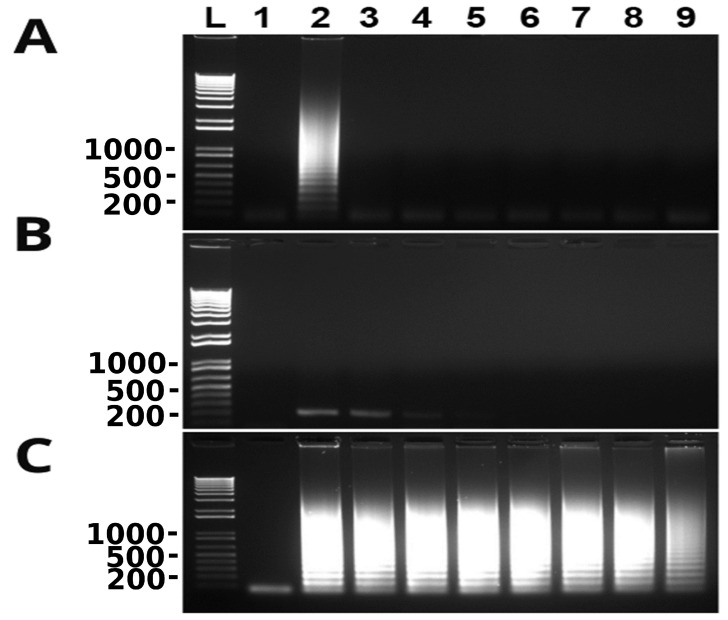
Limit of detection in spiked saliva samples as shown in gel electrophoresis. (**A**) LAMP. (**B**) PCR. Lane 1: nuclease-free water as a negative control; lane 2: positive controls with 100 ng *T*. *tenax* genomic DNA; lanes 3–9: canine saliva spiked with 2 × 10^5^, 2 × 10^4^, 2 × 10^3^, 2 × 10^2^, 2 × 10^1^, 2 × 10^0^ and 2 × 10^−1^ cells, respectively. They were boiled at 100 °C for 30 min with no DNA extraction. (**C**) LAMP using the similarly prepared samples as above that were re-suspended in TE buffer after being washed in PBS. L: 100 bp DNA ladder marker. One of three repeats is presented.

Since *T. tenax* resides in the oral cavity of humans and canines, we next aimed to determine the limit of detection of the newly developed LAMP assay in saliva. We first spiked canine saliva with cultured *T. tenax* using a 10-fold serial dilution ranging from 2 × 10^5^ to 2 × 10^−1^ cells. The cells were boiled at 100 °C for 30 min without DNA extraction and directly used in both LAMP and conventional PCR. The assay showed no amplification in LAMP; however, conventional PCR showed a detection limit of 2 × 10^3^ cells (Figure 5A,B). These data demonstrate that canine saliva contains some inhibitors for LAMP, but not for conventional PCR.

We next investigated whether PBS washed spiked saliva samples can be directly used without DNA extraction in LAMP. Similarly, as mentioned above, a 10-fold serial dilution of cultured *T. tenax* cells was spiked in saliva. The spiked samples were centrifuged, and the cell pellets were resuspended in PBS as wash. The cells were centrifuged once again and then re-suspended in TE buffer. The latter was directly used to perform LAMP. The results showed that the limit of detection was also 0.2–1 cell per LAMP reaction (Figure 5C).

**Figure 6:**
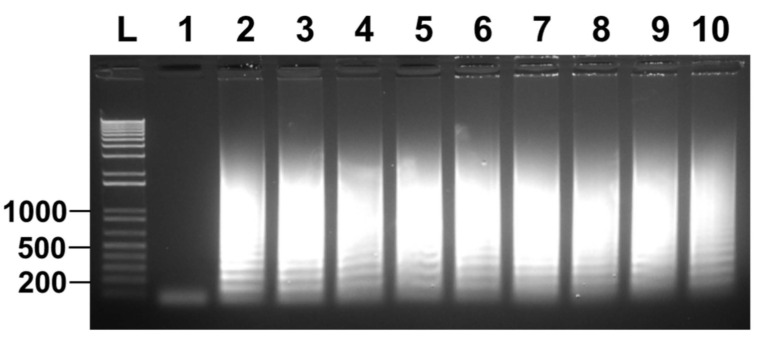
Direct detection of *T. tenax* among clinical samples without prior DNA extraction. Lane 1: nuclease-free water as a negative control; lane 2: positive controls with 100 ng *T. tenax* genomic DNA; lanes 3–10: eight microscopically confirmed trichomonad samples of individually owned pet dogs containing two cells per sample. LAMP results are detected by gel electrophoresis. One of three repeats is presented.

Finally, we were interested in whether canine samples can be directly used in detection of *T. tenax* without prior DNA extraction. We have been collecting oral swabs along the gumline of client owned pet dogs presented to RUSVM Veterinary Clinic. From 44 samples collected eight were positive for trichomonad protozoa by microscopy after initial culture in Diamonds’ media. The cells were pelleted by centrifugation after being washed in PBS and were immediately frozen at −80 °C. Cells from each of these frozen samples were picked up with a pipette-tip and re-suspended in PBS and quantified. They were then diluted in TE buffer and directly used in LAMP. As shown in Figure 6, all eight canine samples were detected as positive for *T. tenax.* Collectively, our data convincedly showed that clinical oral swabs can be directly used in the detection of *T. tenax* by the newly developed LAMP without prior DNA extraction. Therefore, it can be used at the point-of-care of both medical and veterinary clinics.

## 4. Discussion

The LAMP assay developed in this study to detect *T. tenax* is a fast and easy method to perform. It has been previously shown that LAMP amplification takes place in 30 min if loop primers are used to accelerate the reaction [24]. The optimized time for the newly developed LAMP assay is 60 min. However, the results also showed that amplification using loop primers took place in as little as 30 min (Figure 1B), which is in agreement with the previous observations [24].

The LAMP assay designed has a 1000-fold lower LD compared to conventional PCR when both methods targeted the same fragment of ITS and 5.8S rRNA gene of *T. tenax*, i.e., 10 fg vs. 10,000 fg of *T. tenax* DNA, which is similar to several other studies [16,25]. Many other studies on LAMP have reported LD between 10 and 100 fold lower in comparison to PCR [17,18,26]; some others have reported similar LD between LAMP and PCR [27,28]. Further, LD of the conventional PCR varies depending upon its target sequence. It is 100 fg of genomic DNA of *T. tenax* targeting the 18S rRNA gene [1] although data are not available in the literature for other targets such as β-tubulin [4]. Ten times greater analytical sensitivity of the current study targeting the ITS and 5.8 s rRNA gene by LAMP than the previous one targeting the 18S rRNA gene by conventional PCR is likely due to the high copy nature of the former although the exact copy number is not known at present. The LAMP assay was not only sensitive but was also found to be specific, as illustrated in Figure 3. There was no cross-reactivity seen with any of the microorganisms used (*S. pyogenes*, *S. aureus*, *E. coli*, *E. faecalis* and *C. albicans*), or even with *T. vaginalis*, a close relative of *T. tenax*. This high specificity of LAMP is the result of 4–6 primers targeting 6–8 regions of the target DNA [15]. Additionally, after performing a multiple sequence alignment using the software CLUSTAL among *T. tenax*, *T. vaginalis* and *T. brixi* (Supplementary Materials Figure S1) we predict that our newly developed LAMP would be able to distinguish *T. tenax* from *T. brixi* as well. We could not confirm this prediction due to lack of *T. brixi* DNA. The latter is a new trichomonad species recently found in dogs and cats in Europe [13].

Since oral swabs are normally collected from the oral cavity for the detection of *T. tenax*, in this study spike saliva samples were evaluated. A 10-fold serial dilution from 10^5^ to 10^−1^ of *T. tenax* cells was undertaken using saliva, these samples were boiled and directly used to perform LAMP. The results showed no amplification for any of the dilutions, only the positive control (Figure 5A). However, when the samples were used to perform PCR, amplification was seen for cell concentrations as low as 10^3^ cells (Figure 5B). This means that there is a possibility that the LAMP assay is inhibited by saliva. This was also observed in Figure 4A when PBS was serially spiked with *T. tenax*, the LAMP reaction was also inhibited by PBS as no amplification was seen for any of the dilutions. This observation agrees with an earlier discovery that biological samples such as blood and urine, and PBS, can inhibit LAMP reactions [29]. Until now, there are no reports on saliva inhibiting LAMP reactions. Serially diluted spike TE buffer samples, on the other hand, showed a detection limit as low as 0.2–1 cell per reaction (Figure 4B). Furthermore, 10-fold serially diluted saliva samples were washed with PBS and the cells were re-suspended in TE buffer. The results show a detection limit of 0.2–1 cell per reaction (Figure 5C). These data collectively show that the present LAMP assay developed to specifically detect *T. tenax* does not need a DNA extraction or cell boiling step as samples in TE buffer can be used directly in LAMP. This makes this assay even more rapid and easier to perform than other LAMP assays that depend on DNA extraction or a cell boiling step. Eight oral samples collected from different canines were cultured and the cells were diluted to two cells per reaction to perform LAMP. All the samples were positive for *T. tenax* (Figure 6). The low limit of detection and specificity exhibited by this newly designed LAMP assay is possibly a result of its six primers targeting eight different regions of the target DNA, making it possible to amplify even a fragment of the target genomic DNA as long as it is recognized by these primers.

In comparison to traditional techniques such as culture and microscopy, LAMP has been proven to be far more sensitive and specific. The advantage that LAMP has over culture is that cultures take 3 days or more to grow and demonstrate the parasite, it is labor intensive and has a high-cost due to equipment and reagents required. LAMP is a simple, rapid and cheaper diagnostic test compared to culture. Results for LAMP can be obtained in one hour, the reagents are cheap, and it does not require expensive equipment and it is not labor intensive. Microscopy is considered the gold standard in diagnosing trichomonad protozoa. Unlike culture, microscopy is simple, cheap and does not require as much labor as culture. However, microscopy requires well-trained personnel to properly identify the parasite of interest. The major disadvantage of direct microscopy is that it has very low sensitivity, hence why samples are often cultured first and then identified by microscopy. LAMP on the other hand is far more sensitive as well as specific compared to culture, microscopy and PCR.

Although LAMP has many advantages, there are some disadvantages as well. Due to the high sensitivity of LAMP in comparison to PCR, reports have shown that LAMP reactions have a higher possibility of getting contaminated or producing false-positive results [14,30,31]. Despite the disadvantages, LAMP remains a powerful molecular diagnostic tool for the rapid detection of *T. tenax* and other infectious pathogens in countries with limited laboratory resources. Recently, the newly developed LAMP described here has been directly used to detect *T. tenax* in a dozen mouth samples collected from owned dogs on St. Kitts using a cotton swap. The samples were immediately preserved in 70% ethanol upon collection in the field. They were tested in a laboratory after being resuspended in TE buffer followed by washing in PBS without DNA extraction. Several samples were detected as positive for *T. tenax*, which unequivocally demonstrates that the LAMP presented here is capable of diagnostically detecting *T. tenax* at point-of care (unpublished data). Alternatively, a clinical sample of human or canine origin, or of other mammals as sources, can be put into the physiological saline upon collection. The sample is then centrifuged, and the pellet is resuspended in TE butter, of which 2 µL can be directly applied to LAMP mixture for testing. In short, this newly developed LAMP assay has shown how applicable and convenient it is for use at point-of-care of both medical and veterinary clinics. The newly developed assay does not require DNA extraction, cells can be directly applied to perform LAMP and the results can be obtained within one hour. At point-of-care, this LAMP assay could be a powerful diagnostic tool to effectively and rapidly detect *T. tenax.*

## 5. Conclusions

The LAMP assay developed in this study to detect *T. tenax* is rapid, specific, sensitive and easy to perform. The assay designed is an affordable and powerful diagnostic test for the detection of *T. tenax* at the point-of-care in medical and veterinary clinics in both developed and developing countries. Further studies on clinical samples are needed to validate its diagnostic sensitivity and specificity.

## Figures and Tables

**Table 1:** Loop-mediated isothermal amplification (LAMP) primer sequence for detection of *T. tenax*.

Name of Primer	Primer Sequence (5′–3′)
FIP (forward inner primer)	GTCATGATGTATGCAACTCCGG-TCCTCACACGATGAAGAACG
BIP (backward inner primer)	GGTTAATCTTTGAATGCAAATTGCG-TGTACTGTTACACGCATGCTTCT
LF (forward loop primer)	ACATTATGCCACGTTCTTCATCG
LB (backward loop primer)	TGCGCTAAACTTGGCTTCGG
F3 (forward outer primer)	AGCAATGGATGTCTTGGC
B3 (backward outer primer)	GCAGACAACGTAAGTTTGT

## Data Availability

All data are presented in the manuscript and the Supplementary Materials.

## Supplementary Materials

The following are available online at https://www.mdpi.com/2076-2607/10/3/594/s1, Figure S1: Alignment of ITS and 5.8S rRNA sequences of *Trichomonas tenax* (U86615), *T. vaginalis* (U86613) and *T. brixi* (KX977528). Loop-mediated isothermal amplification (LAMP) primers are color coded in *T. tenax* sequence.
