# Correction: Matthew et al. A Loop-Mediated Isothermal Amplification (LAMP) Assay Specific to *Trichomonas tenax* Is Suitable for Use at Point-of-Care. *Microorganisms* 2022, *10*, 594

**DOI:** 10.3390/microorganisms11112736

**Published:** 2023-11-09

**Authors:** Maurice A. Matthew, Jevan Christie, Nawu Yang, Chaoqun Yao

**Affiliations:** 1 Department of Biomedical Sciences, Ross University School of Veterinary Medicine, P.O. Box 334, Basseterre 00334, Saint Kitts and Nevis; MMatthew@rossvet.edu.kn (M.A.M.); NawuYang@students.rossu.edu (N.Y.); 2 One Health Centre for Zoonosis and Tropical Veterinary Diseases, Ross University School of Veterinary Medicine, P.O. Box 334, Basseterre 00334, Saint Kitts and Nevis; jevanchristie@gmail.com; 3 Department of Clinical Sciences, Ross University School of Veterinary Medicine, P.O. Box 334, Basseterre 00334, Saint Kitts and Nevis

In the original publication [1], there was a mistake in Figures 1–6, the marker labels of the DNA ladder in all Figures 1–6 were a little off.

To the correct version appears below. 

**Figure 6:**
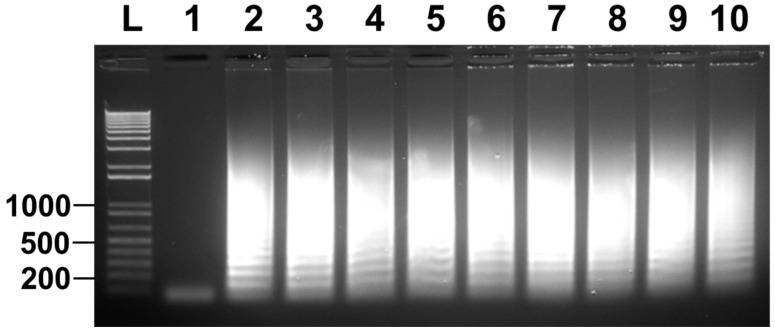
Direct detection of *T. tenax* among clinical samples without prior DNA extraction. Lane 1: nuclease-free water as a negative control; lane 2: positive controls with 100 ng *T. tenax* genomic DNA; lanes 3–10: eight microscopically confirmed trichomonad samples of individually owned pet dogs containing two cells per sample. LAMP results are detected by gel electrophoresis. One of three repeats is presented.

The marker labels of DNA ladder in Figures 1–6 should be the same as those in the corrected Figure 6 showed above.

In the original publication [1], there was a mistake in Table 1, as published.

“LP (backward loop primer)” in the fourth line of Table 1 was wrong. 

The corrected content appears below.

**Table 1:** Loop-mediated isothermal amplification (LAMP) primer sequence for detection of *T. tenax*.

Name of Primer	Primer Sequence (5′–3′)
FIP (forward inner primer)	GTCATGATGTATGCAACTCCGG-TCCTCACACGATGAAGAACG
BIP (backward inner primer)	GGTTAATCTTTGAATGCAAATTGCG-TGTACTGTTACACGCATGCTTCT
LF (forward loop primer)	ACATTATGCCACGTTCTTCATCG
LB (backward loop primer)	TGCGCTAAACTTGGCTTCGG
F3 (forward outer primer)	AGCAATGGATGTCTTGGC
B3 (backward outer primer)	GCAGACAACGTAAGTTTGT

There was an error in the original publication [1]. “LF, and LP (Table 1).” in the fourth line of Section 2.2. LAMP Reaction on page 2 was wrong. 

A correction has been made to Section 2.2. LAMP Reaction on page 2: 

The LAMP primers targeting the ITS and 5.8S rRNA gene of *T. tenax* (GenBank Accession No. U86615) were designed using the software Primer explorer V.5 (http://primerexplorer.jp (accessed on 28 February 2020). These include FIP and BIP, F3 and B3, LF, and LB (Table 1). Multiple sequence alignment using software CLUSTAL 1.2.4 (Clustal Omega; https://www.ebi.ac.uk/Tools/msa/clustalo/ (accessed on 28 February 2020) was carried out on the closely related protozoan *T. vaginalis*, to check for specificity.

The authors state that the scientific conclusions are unaffected. This correction was approved by the Academic Editor. The original publication has also been updated. 
