# Supplementary material for: A Loop-Mediated Isothermal Amplification (LAMP) Assay Specific to Trichomonas tenax Is Suitable for Use at Point-of-Care
Source: Microorganisms. 2022 Mar 10;10(3):594. doi: 10.3390/microorganisms10030594 (PMC8949391; doi:10.3390/microorganisms10030594)
Supplement: Supplementary file 1 [file microorganisms-10-00594-s001.zip › microorganisms-1574265-supplementary.pdf]

```
KX977528      ----------------------atcagttctagttataataactaataccaacttctttt   38
U86615        cggtaggtgaacctgccgttggatcagttctagtttaataactaataccaacttct---t   57
U86613        cggtaggtgaacctgccgttggatcagttctagttttaataactaacaccaacttc---t   57
                                    *************  *  *   *   * *  *     *

KX977528      taattaaatcacaaaacaatacaaatttaaataactaacttcatcaaaaaatcaagtctc   98
U86615        tttattaatcaaaaactaatacaattataaataactaact-tcatcaaaaatcaagtctc   116
U86613        tttattaaacaaaaaccaata-caaaattaaaaactaact-tcatcaaaaaccaagtctc   115
              *   * ** ** ***  ****  *   * ** ********      ***** ********

KX977528      taagcaatggatgtcttggctcctcacacgatgaagaacgtggcataatgtgttaagtaa   158
U86615        taagcaatggatgtcttggctcctcacacgatgaagaacgtggcataatgtgttaagtaa   176
                F3                FIP(F2) LF
U86613        taagcaatggatgtcttggctcctcacacgatgaagaacgtggcataatgtgttaagtaa   175
              ************************************************************

KX977528      ccggagttgcaaacatcatgacaggttaatctttgaatgcaaattgcgctttccc-ggct   217
U86615        ccggagttgcatacatcatgacaggttaatctttgaatgcaaattgcgctaaacttggct   236
              FIP(F1c)               RIP(B1)             LP
U86613        ccggagttgcaaacatcatgacaggttaatctttgaatgcaaattgcgctaaactcgatc   235
              *********** **************************************   *  *

KX977528      tcggccgaggagcatgcgtgtaacagtacaacattaattataataattcttattctaagc   277
U86615        tcggctgagaagcatgcgtgtaacagtacaacataatttataataattcttattctaagc   296
                     RIP(B2)
U86613        tcggtcgagaagcatgggtgtgacagtactacatctttttataataattcttattctaagc   295
              ****  *** ****** **** ******* ****   ***********************

KX977528      gaataagtaaataaaattataagacaaacaatacgttgtctgctatacgca---------   328
U86615        gaataagtaaataaatt-ataagacaaact-tacgttgtctgctatacgcaggaagaccc   354
                                     B3
U86613        gaataagtaaataatat-ataagacaaacaacacgtagtctgccatacgcaggaagaccc   354
              **************  * ***********   **** ****** *******

KX977528      --------------      328
U86615        gctgaactgaagca      368
U86613        gctgaactgaagca      368
```

Figure S1. Alignment of ITS and 5.8S rRNA sequences of *Trichomonas tenax* (U86615), *T. vaginalis* (U86613) and *T. brixi* (KX977528). LAMP primers are color coded in *T. tenax* sequence.
